# Novel Gene Signature Reveals Prognostic Model in Acute Myeloid Leukemia

**DOI:** 10.3389/fgene.2020.566024

**Published:** 2020-10-28

**Authors:** Ying Qu, Shuying Zhang, Yanzhang Qu, Heng Guo, Suling Wang, Xuemei Wang, Tianjiao Huang, Hong Zhou

**Affiliations:** Department of Hematology, The Second Affiliated Hospital of Qiqihar Medical College, Qiqihar, China

**Keywords:** AML, modularization, machine learning, prognostic model, FLT3

## Abstract

**Background:**

Acute myeloid leukemia (AML) is a clonal malignant disease with poor prognosis and a low overall survival rate. Although many studies on the treatment and detection of AML have been conducted, the molecular mechanism of AML development and progression has not been fully elucidated. The present study was designed to pursuit the molecular mechanism of AML using a comprehensive bioinformatics analysis, and build an applicable model to predict the survival probability of AML patients in clinical use.

**Methods:**

To simplify the complicated regulatory networks, we performed the gene co-expression and PPI network based on WGCNA and STRING database using modularization design. Two machine learning methods, A least absolute shrinkage and selector operation (LASSO) algorithm and support vector machine-recursive feature elimination (SVM-RFE), were used to filter the common hub genes by five-fold cross-validation. The candidate hub genes were used to build the predictive model of AML by the cox-proportional hazards analysis, and validated in The Cancer Genome Atlas (TCGA) cohort and ohsu cohort, which were reliable in the experimental verification by qRT-PCR and western blotting in mRNA and protein levels.

**Results:**

Three hub genes, FLT3, CD177 and TTPAL were used to build a clinically applicable model to predict the survival probability of AML patients and divided them into high and low groups. To compare the survival ability of the model with the classical clinical features, we generated the nomogram. The model displayed the most risk points contrast to other clinical characteristics, which was compatible with the data of cox multivariate regression.

**Conclusion:**

This study reveal the novel molecular mechanism of AML, and construct a clinical model significantly related to AML patient prognosis. We showed the integrated roles of critical pathways, hub genes associated, which provide potential targets and new research ideas for the treatment and early detection of AML.

## Introduction

AML is a devastating hematological malignancy. Differentiation arrest and unscheduled proliferation of immature cells of myeloid lineage are characteristic of this disease (Hansrivijit et al., 2019). A variety of chemotherapy regimens, biological agents, and stem cell transplantation are the main treatment options for AML (Liu et al., 2019a,b; Zhou et al., 2019). However, chemotherapy drug toxicity may lead to acute and life-threatening complications. Compared with standard chemotherapy, allogeneic stem cell transplantation is a suitable method to reduce the risk of recurrence of AML, but also increase the risk of serious complications. Although continuously improved, the traditional method of treatment does not lead to a complete cure or an ideal duration of survival for AML in clinical practice (Manola et al., 2013). Genomics, proteomics and bioinformatics analysis methods have been used to develop new personalized treatment strategies, study of the functions of related biomolecules, and collection of information on emerging trends in genome matching of clinical data are effective methods to improve the prognosis of patients (Wang et al., 2015; Bret et al., 2016; Cai and Levine, 2019). Although many studies have analyzed genome variation in AML, the association between genome variation and molecular mechanism of AML is still unclear. Therefore, a comprehensive study of AML was urgent.

In this study, we aimed to explore the molecular mechanism of AML using a comprehensive bioinformatics analysis, and construct a clinical model to identify survival associated hub genes of AML patients. We initially performed the function annotation and modularization of gene differential expression; we then filtered candidate hub genes in GEO training cohort using machine learning algorithm with five-across validation and validated those hub genes in TCGA and ohsu cohort. We also hope that the results of this study can help us identify key pathways and genes related to AML, and provide possible targets and new research ideas for the treatment and early detection of AML.

## Materials and Methods

### Sample Collection

The bone marrow of AML samples and non-leukemia samples were collected from 24 patients at The Second Affiliated Hospital of Qiqihar Medical University. Two independent pathologists made the diagnosis of AML and assessed the samples. Patients characteristics were summarized in Supplementary Table 1.

### Microarray Data Source and Pre-processing

The gene expression profiles of AML were obtained from three data sets, GSE6891, GSE10358 and GSE15061 of the NCBI GEO database, which are based on the Affymetrix HT HG-U133A and HG-U133A 2.0 Array. A total of 223 biochips from AML patients were analyzed, including 154 AML tumor samples and 69 non-leukemia samples. The raw data of the three datasets were downloaded from GEO, and the R package, Simpleaffy, was used for Affymetrix quality control and data analysis (Yu et al., 2010). Annotations were made using gene symbols from each respective platform annotations. Then, expression data from all 223 samples were included into a united gene expression matrix. The mean value of gene expression was used in multiple probes sets with a single gene symbol. Batch correction were performed before the next analysis was conducted using combat method in sva R package (Johnson et al., 2007; Leek et al., 2012).

### Functional Analysis

The limma package was used to identify differentially expressed genes (DEGs) (Ritchie et al., 2015), then GO and KEGG analysis were performed using clusterProfiler (Yu et al., 2012). The log2(fold change) > 1 and BH-adjusted *p* value < 0.05 were filtered as the statistically significant. GSEA was utilized to deeply analyze the variation in biological functional and pathways between AML and non-leukemia samples (Subramanian et al., 2005).

### Module Analysis Using WGCNA Based on PPI Network

To modularize the biological variation in AML, the WGCNA package was used for the co-expression analysis of the DEGs (Langfelder and Horvath, 2008). Then, the data was superimposed onto the PPI database of STRING (Szklarczyk et al., 2015). The co-expression analysis clusters were delineated using the dynamic tree cut package with the minimum height for each module set at 0.2 (Langfelder et al., 2008). The trend of each module was based upon eigengene, and the members of the module were collected through Pearson correlation from among DEGs and their interactors. Moreover, a topological overlapping matrix was also utilized to filter the PPI network (Ravasz et al., 2002). Finally, individual modules were annotated using cluster Profiler (Yu et al., 2012) and were visualized in Cytoscape (Shannon et al., 2003).

### Construction the Predictive Model of AML

Firstly, A least absolute shrinkage and selector operation (LASSO) algorithm and support vector machine-recursive feature elimination (SVM-RFE) were used to filter the hub genes by five-fold cross-validation, respectively (Tibshirani, 1996; Huang et al., 2014). Then, we combined the result from LASSO and SVM-RFE algorithms to filter the common hub genes. Finally, the cox-proportional hazards analysis was performed using glmnet R package, and picked up the risk associated hub genes that were more than 900 times in 1000 repetition (Friedman et al., 2010; Xu et al., 2017). X-tile 3.6.1 software (Yale University, New Haven, CT, United States) was employed to decide the best cutoff for AML patients categorized as low risk and high risk. The prophetic capacity of the prognostic model was assessed by the log-rank test and Kaplan-Meier survival analysis in GEO training cohort, TCGA testing cohort and Ohsu testing cohort.

### Assessment of Nomogram Performance

To predict the survival ability of 1, 3, and 5 year of AML patients, we performed the nomogram analysis depend on the results of multivariate analysis including age, gender, race, chemotherapy status, radiation therapy status, gene fusion and risk type. Moreover, the calibration plot was used to assess the proportion of the predicted probabilities against the observed ones.

### Quantitative Real-Time PCR (qRT-PCR)

qRT-PCR was used to verify the results. The total RNA of AML samples and non-leukemia samples were extracted using TRIzol reagent. The genes of interest were then quantified through qRT-PCR using a One-Step qPCR Kit (Invitrogen, United States), which was executed on a CFX ConnectTM Real-Time System (BIO-RAD, United States), according to the manufacturer’s instructions. The results were analyzed using 2^–ΔΔ*CT*^ method, with GAPDH as a reference gene (Livak and Schmittgen, 2001). The primer sequences of the target genes are shown in Supplementary Table 2.

### Western Blotting Analysis

The tissues were lysed, and total protein was quantified using the Pierce^TM^ Detergent Compatible Bradford Assay Kit (Thermo Scientific). 20 μg of protein from each sample was used for SDS-PAGE. After transferring the sample onto a PVDF membrane, the blot was incubated with indicate antibodies. All antibodies were purchased from CST: CD177 (ab203025, Abcam), TTPAL (ab103740, Abcam), FLT3 (#3462, CST), and GAPDH (#5174, CST).

### Statistical Analysis

All experiments were performed in triplicate, at the least. For analyses between two groups, the student’s *t* test was leveraged for the comparison of tumor tissue with adjacent tissue. Data are presented as mean SDs, except when indicated otherwise. A *p* value < 0.05 was considered to be statistically significant.

## Results

### Identification of Differential Expression Genes (DEGs) and Functional Variation

**FIGURE 1:**
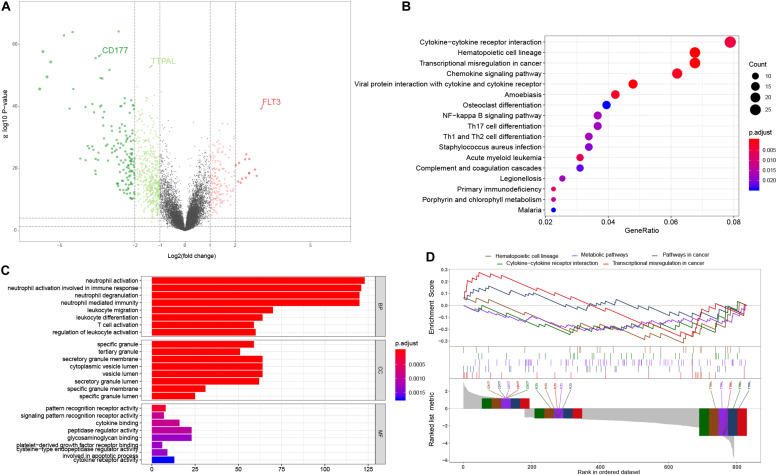
Functional analysis. **(A)** The volcano plot of the 1,084 DEGs between AML tumor samples and non-leukemia samples. Genes know to be upregulated and downregulated are displayed with different colors. Three hub genes chosen for model construction are indicated; **(B)** The KEGG pathway enrichment analysis showed that transcriptional misregulation in cancer, hematopoietic cell lineage, cell cycle, and TH1, 2, 17 cell differentiation were the most significantly affected phases in AML; **(C)** The most enriched GO targets were involved in neutrophil activation, neutrophil degranulation, neutrophil activation involved in immune response, neutrophil mediated immunity and leukocyte migration; **(D)** The GSEA results of AML patients and non-leukemia tissues performed on all genes at transcription level. Three hub genes chosen for model construction are indicated.

We used the limma package to screen out DEGs from 154 AML samples and 69 non-leukemia samples. The inclusion criteria of the DEGs was an absolute log2FC > 1 and the BH-adjusted *p* value < 0.01 was used as the statistical filter conditions. 1,084 DEGs, including 202 significantly upregulated genes and 882 significantly downregulated genes, were obtained. The volcano plot of DEGs is shown in Figure 1A.

In order to further analyze DEGs, we explored the functional variation between the two groups using the cluster Profiler package. 107 GO terms were identified with the BH-adjusted *p* value < 0.01. The GO SemSim package was used to remove duplicate terms, keeping only one representative term, which resulted in 49 unique GO terms (Yu et al., 2010). The results of the GO analysis showed that the most enriched GO targets were involved in neutrophil activation, neutrophil degranulation, neutrophil mediated immune response and leukocyte migration (Figure 1B). The KEGG pathway enrichment analysis showed that transcriptional misregulation in cancer, hematopoietic cell lineage, cell cycle, and TH1, 2, 17 cell differentiation were the most significantly affected phases in AML (Figure 1C). These results complemented the results of the GO enrichment analysis. In order to further verify the relationship between the phenotype and functionally differentiated genes, we performed GSEA analysis on all genes at transcription level. The transcripts of AML were found to be remarkably associated with downregulated genes related to three pathways (Figure 1D).

### Integrative Network Analysis Reveals New Functional Modules

**FIGURE 2:**
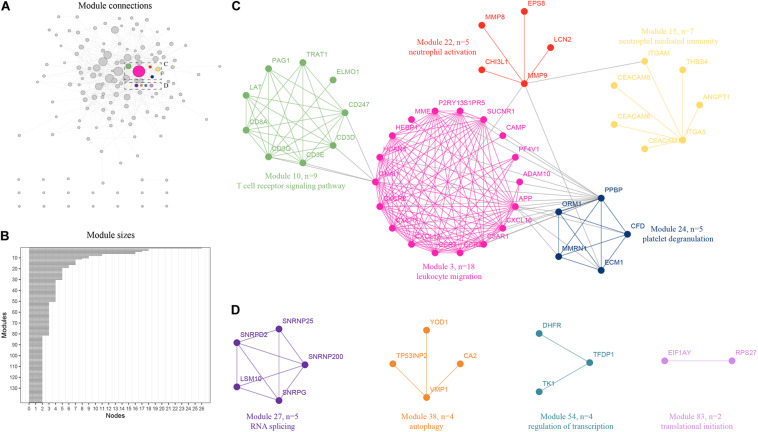
Expression profiling of proteome reveals co-expression clusters and functional modules in AML. **(A)** Distribution of 120 out of 143 modules. Each node represents the individual module and their interactions by the module size. Edges connect modules that share PPIs. Boxed modules are further enlarged in C and D; **(B)** 143 modules with the number of proteins ranging from 2 to 25 were identified; **(C)** Module 3, 10, 15, 22, and 24 were notably enriched in hematopoietic system related progression showing the protein names and representative functional terms; **(D)** Module 27 was found to be involved in RNA splicing, module 38 was found to be involved in autophagy, module 54 was found to be involved in the regulation of transcription, while module 83 was found to be involved in translational initiation.

**FIGURE 3:**
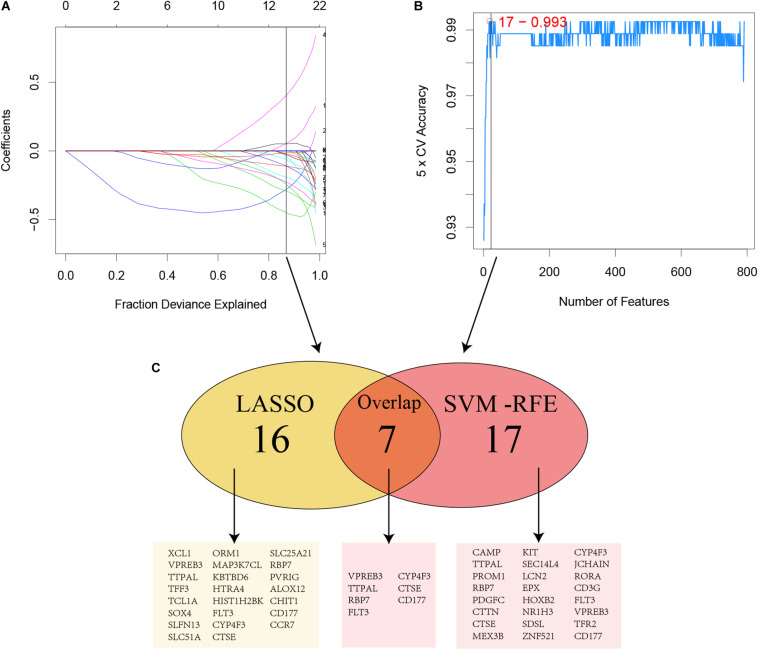
Two algorithms were performed for hub genes selection. **(A)** LASSO; **(B)** SVM-RFE; **(C)** Common genes selected by two algorithms.

An integrative analysis method was used to model the dynamics of proteome changes upon cancer progression, as previously described (Tan et al., 2017). We applied WGCNA to all DEGs to cluster the correlative proteins that had similar molecular functions or biological processes (Jansen et al., 2002). Later, these proteins were superimposed onto the PPI network to identify the functional modules. As a result, we identified 143 modules with the number of proteins in each ranging from 2 to 25 (Figure 2A), and 122 of these modules were highly interconnected by their members (Figure 2B). Each module was annotated using known functional terms or signaling pathways. We found that many modules, including module 3, 10, 15, 22, and 24 (Figure 3C), were notably enriched in hematopoietic system related progression. In addition, module 27 was found to be involved in RNA splicing, module 38 was involved in autophagy, module 54 was involved in the regulation of transcription, while module 83 was involved in translational initiation (Figure 3D). In summary, the progression of AML involves the balanced regulation and extensive reprogramming of mutually connected functional modules.

### Construction, Validation and Assessment of the Predictive Model of AML

**FIGURE 4:**
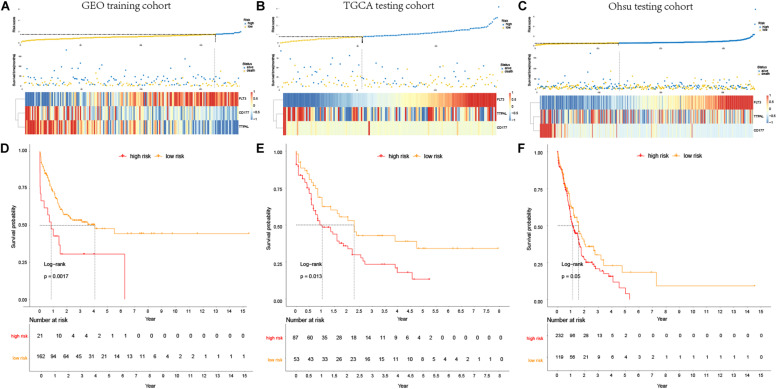
Prognostic analysis of the predictive model. **(A–C)**. Association between the risk score (upper) and the expression of three prognostic hub genes (bottom) is displayed in GEO training cohort, TCGA testing cohort and Ohsu testing cohort; **(D–F)**. Kaplan-Meier survival showed OS was significantly higher in the low-risk score subgroup than in the high-risk score subgroup in GEO training cohort, TCGA testing cohort, and Ohsu testing cohort.

**FIGURE 5:**
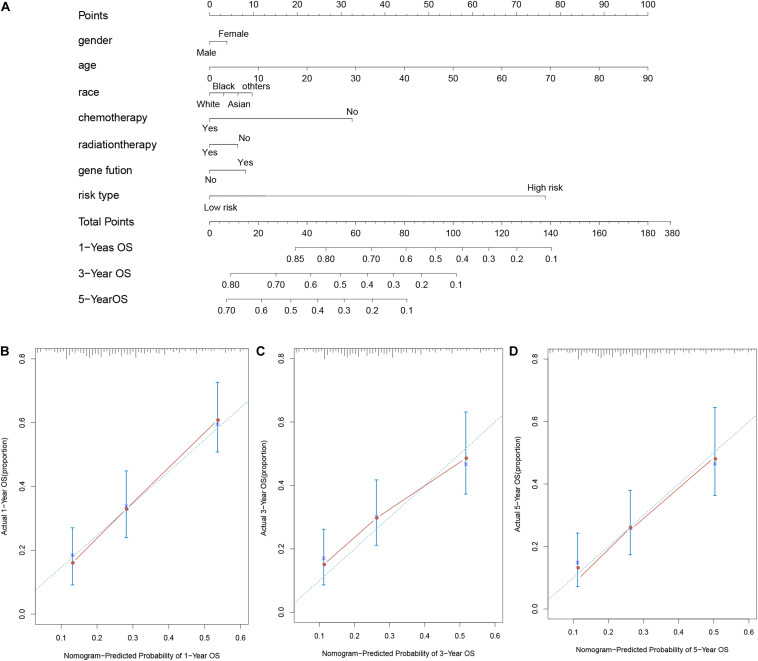
AML survival nomogram. **(A)** Nomogram for predicting the probability of 1, 3, and 5 years OS for AML patients; **(B–D)**. Calibration plot of the nomogram for predicting the probability of OS at 1, 3, and 5 years.

For considering the variation between AML patients and healthy people, we aimed to estimate the predictive potential of DEGs. After differential expression analysis, we get 1084 DEGs in AML patients. Next, we performed two distinct machine learning algorithms, the LASSO and SVM-RFE, to screen the most significant DEGs for building the prognostic model. By the LASSO algorithm, we validated a set of 16 hub genes. And we also chose a set of 17 hub genes using the SVM-RFE algorithm. After integrating the hub genes from the LASSO and SVM-RFE algorithms, we obtained 40 hub genes with 7 hub genes identified simultaneously by the two machine learning algorithms with five-fold cross-validation. In detail, the training set was randomly divided into five equal portions; then, during each of the five iterations, we first performed the LASSO and SVM-RFE as the feature selection method on 4/5 of the training data and trained the classifiers with the selected features. Next, we applied the trained classifiers to the remaining 1/5 of the training data for prediction. Finally, the predictions from all five iterations were then combined and compared with the truth. The 7 significant hub genes are VPREB3, CYP4F3, TTPAL, CTSE, RBP7, CD177, and FLT3 (Figures 3A–C). Then, the cox-proportional hazards analysis was used to stratify the AML patients in to high and low risk subgroups. We established the predictive model by calculating the risk score to predict the ability of survival in GEO training cohort (Figure 4A) (risk score = normalized expression level of FLT3 ^∗^ 0.261 + normalized expression level of CD177 ^∗^ 0.327 - normalized expression level of TTPAL ^∗^ 0.555). The cutoff point of high and low patients was obtained using X-tile software. Figures 4B,C showed the predictive ability of the prognostic model in TCGA and Ohsu testing cohort, respectively. The results of Kaplan-Meier survival analysis were shown in Figures 4D,E. Moreover, to identify if FLT3, CD177 and TTPAL genes influence the AML prognosis independently, we performed survival analysis and found that these three hub genes were involved in the prognosis of AML independently or in the established model. Finally, we created a nomogram to predict the 1, 3, and 5 years overall survival for AML patients. The model displayed the most risk points contrast to other clinical characteristics, which was compatible with the data of cox multivariate regression (Figure 5A). Finally, the calibration plot was used to assess the consistency between the prediction and the observation. As expected, the results found to be near to the ideal curve (Figure 5B).

### Experimental Verification of Candidate Genes in mRNA and Protein Levels

**FIGURE 6:**
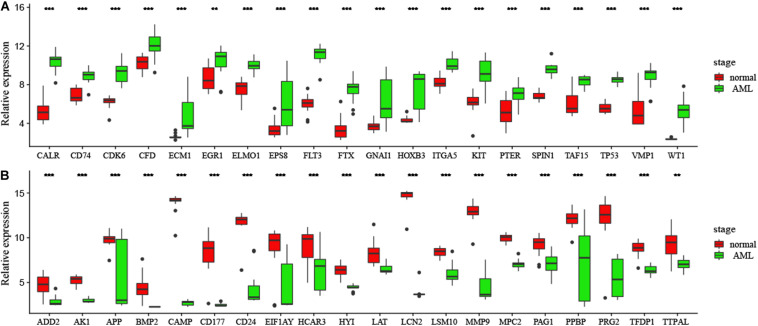
**(A,B)** Validation of DEGs by qRT-PCR. Boxplots indicate the medians and dispersions of 40 AML and normal samples. *P*-values are counted by student’ *t* test, *p < 0.05, **p < 0.01, ***p < 0.001.

In order to confirm DEGs, the total RNA of 24 paired AML samples were isolated for qRT-PCR validation. 40 target DEGs were selected, as shown in Figure 6. The DEGs were successfully validated and showed a good correspondence with the results of the transcriptome analysis, indicating precise and reliable microarray results.

**FIGURE 7:**
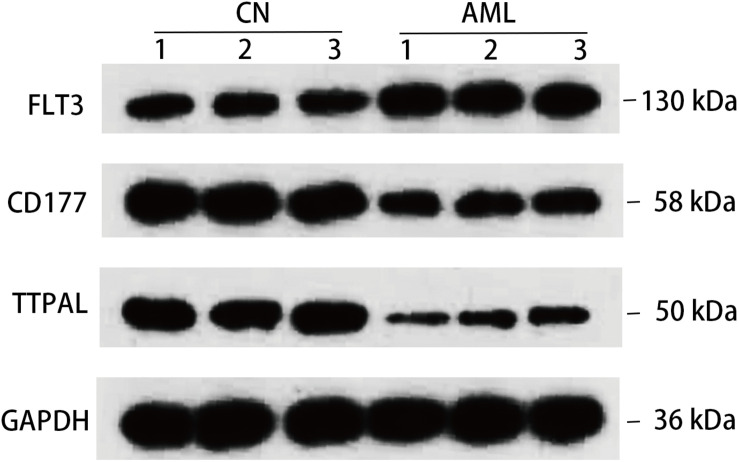
Detection in protein level. Western blotting detection of indicated protein. Lysates from three pairs of AML and normal samples were subjected to western blotting with antibody to, FLT3, CD177, TTPAL, and GAPDH. GAPDH is a reference gene.

At the same time, we confirmed 3 hub genes at protein level, FLT3 protein expression levels were all found to be upregulated in AML, and CD177 and TTPAL were downregulated in AML, which is consistent with the results of the qRT-PCR (Figure 7). One of the most widely studied gene in the hematopoiesis of AML is FLT3. FLT3 is class III receptor tyrosine kinases that play a crucial role in hematopoiesis (Reilly, 2002). The pathogenesis of several malignant tumors are associated with the overexpression of FLT3 (Fassunke et al., 2010). In particular, the FLT3 genes have been intensively studied in childhood AML (Liang et al., 2002; Boissel et al., 2006). CD177 is mostly expressed in neutrophils, and is upregulated in tumor tissues of patients with colitis associated cancer (CAC). CD177 has been proven to predict the benign prognosis of colorectal cancer (Bai et al., 2017; Zhou et al., 2018).

## Discussion

AML is a clonal malignant disease with a poor prognosis and low overall rate of survival. It originates from hematopoietic bone marrow primordial cells. Immature leukocytes grow rapidly and interfere with the production of normal blood cells. The median survival time of AML patients is only 5–10 months (Hansrivijit et al., 2019). The overall survival rate (OS) of traditional treatment (chemotherapy and stem cell transplantation) for AML is low, and chemotherapy is easily accompanied by complications, while stem cell transplantations are high in cost, with a risk of being rejected (Vaughn et al., 2019). The molecular mechanism of AML development and progression is not fully understood, and it is particularly important to find new targets and strategies for individualized therapy.

In this study, we combined three datasets from Gene Expression Omnibus (GEO) as GEO cohort, including 377 AML samples and 69 non-leukemia samples. The combat algorithm in sva R package was used to remove the batch effect. By GO and KEGG analyses, we found that dysfunctions of AML patients were primarily enriched in cytokine-cytokine receptor interaction, transcriptional mis-regulation in cancer, chemokine signaling pathway, and neutrophil related functions, such as neutrophil activation, neutrophil degranulation, neutrophil mediated immunity and so on. Moreover, traditional strategies for gene expression analysis have focused on identifying individual genes that exhibit differences between two or more states of interest. Some specific pathways might be significantly affected while changes in expression of individual genes are relatively subtle. To address this puzzler, we performed GSEA using MSigDB (c5.bp.v6.2.symbols.gm) as reference gene set. The results of GSEA proved the previous conjecture and in good agreement with GO and KEGG results.

In addition to the function annotation of gene differential expression, we also explored the gene co-expression and PPI network based on WGCNA and STRING database using modularization design. Modules were generated from hierarchical cluster tree algorithm and topological overlapping matrix, and then functionally annotated (Ravasz et al., 2002; Langfelder et al., 2008). In this approach, the intricate regulatory networks were facilitated into simple and easy modules, which were conducive to ascertain the connections of hub genes in the biological processes. From modularization analysis, we found that the progression of AML was related with balanced regulation and extensive reprogramming of mutually connected functional modules, such as leukocyte migration, T cell receptor signaling (TCR) pathway, autophagy and RNA splicing. The function of the autophagy in cancer, as a driver of oncogenic transformation or inhibitor of tumor progression, remains a controversial topic. Watson et al. found that hematopoietic stem and progenitor cells possess elevated autophagic flux than mature hematopoietic cells, but the flux of AML cells tends to decrease. This combined with the fact that genes related autophagy were subject to copy number variation (CNV) loss in AML, may imply the connection between decreased autophagy and the progression of AML (Watson et al., 2015). Recently, the dysfunction of gene splicing in AML development and drug resistance have received attention. Several recent studies have emphasized that splicing factor mutations are important drivers of hematological malignancies (de Necochea-Campion et al., 2016; Zhou and Chng, 2017; Tyner et al., 2018). For example, Oncogene Wilms’ tumor gene 1 (WT1) is a target for immunotherapy and biomarker in AML, and a large number of isoforms of WT1 were validated. Among them, +5/ + KTS are the notable variant at prognosis, although the ratio swings (Siehl et al., 2004; Lopotová et al., 2012).

Benefit from the method of machine learning, we established a clinically applicable model to predict the survival probability of AML patients. This model was built in GEO cohort, and validated in TCGA and ohsu cohort. Our results showed that AML patients could be stratified into two subgroups with high or low risks of OS. Kaplan-Meier survival analysis was used to value the prophetic capacity. The clinical features and accuracy of model were assessed in the nomogram and calibration plot. Furthermore, the three hub genes identified by machine learning algorithms were reliable in the experimental verification by qRT-PCR and western blotting in mRNA and protein levels. All these suggested that the conformity strategy was feasible. In addition, the three final hub genes discovered are all novelly associated with cancer, especially FLT3. FLT3 is considered to be a target of treatment for AML, and at present, the development of clinical targets related with FLT3 is very active. FLT3 is characterized by the presence of five immunoglobulin-like motifs within their extracellular section. These motifs are exclusively expressed in hematopoietic cells (Blume-Jensen and Hunter, 2001). FLT3 mutations occur as secondary events during AML clonal evolution (Shlush et al., 2014). FLT3-ITD mutation has a negative impact on the prognosis of AML, only a minority of patients with FLT3-ITD mutation in leukemic blasts are cured through chemotherapy.

Overall, we explored the molecular mechanism that influence the occurrence and development of AML at the genome level using an integrated method, and built a model to predict the survival probability of AML patients in clinical use. We also hope that the results of this study may help to identify critical pathways and genes associated with AML and provide potential targets and new research ideas for the treatment and early detection of AML.

## Data Availability Statement

Publicly available datasets were analyzed in this study. The TCGA database: https://portal.gdc.cancer.gov. The Gene Expression Omnibus: https://www.ncbi.nlm.nih.gov/geo/.

## Ethics Statement

The study protocol was approved by the Ethics Board of the Second Affiliated Hospital of Qiqihar Medical University and complied with the Declaration of Helsinki.

## Author Contributions

HZ designed the study and wrote the manuscript. YiQ analyzed the data. SZ, YaQ, and HG collected the data. YiQ, SW, XW, and TH performed the experiment. All authors read and approved the manuscript.

## Conflict of Interest

The authors declare that the research was conducted in the absence of any commercial or financial relationships that could be construed as a potential conflict of interest.
