# Supplementary figures and images for: Novel Gene Signature Reveals Prognostic Model in Acute Myeloid Leukemia
Source: Front Genet. 2020 Oct 28;11:566024. doi: 10.3389/fgene.2020.566024 (PMC7655922; doi:10.3389/fgene.2020.566024)

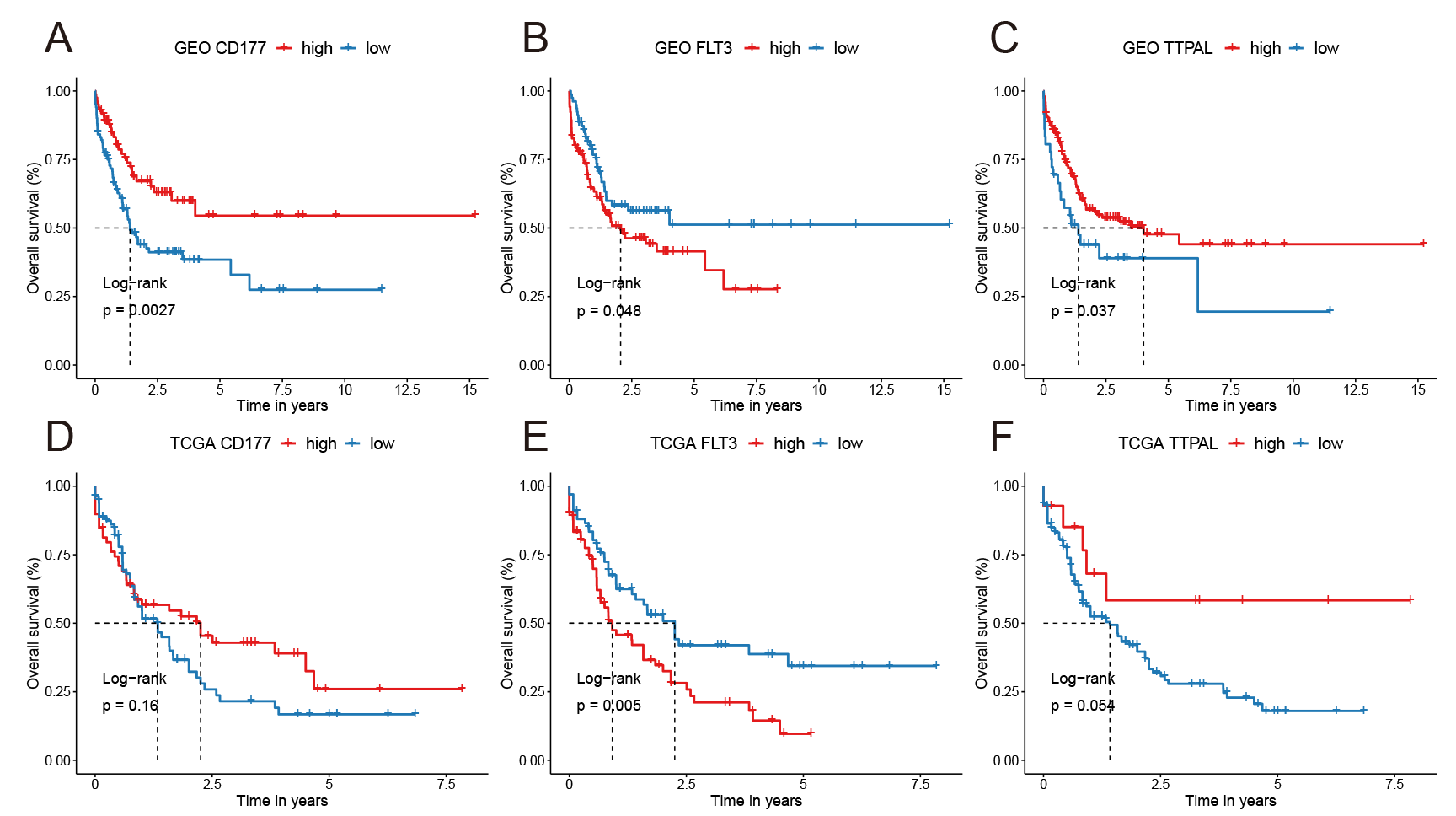

Supplement: Supplementary file 1 [file Image_1.TIF]
